# Factors Influencing the Progression and Direction of Scoliosis in Children with Neurological Disorders

**DOI:** 10.3390/children9010081

**Published:** 2022-01-06

**Authors:** Yeun-Jie Yoo, Jung-Geun Park, Leechan Jo, Youngdeok Hwang, Mi-Jeong Yoon, Joon-Sung Kim, Seonghoon Lim, Bo-Young Hong

**Affiliations:** 1 Department of Rehabilitation Medicine, St. Vincent’s Hospital, College of Medicine, The Catholic University of Korea, Seoul 16247, Korea; yeunjie@catholic.ac.kr (Y.-J.Y.); jgp0123@naver.com (J.-G.P.); joychan85@hotmail.com (L.J.); allogen@naver.com (M.-J.Y.); svpmr@chol.com (J.-S.K.); limseonghoon@gmail.com (S.L.); 2 Paul H. Chook Department of Information Systems and Statistics, Baruch College, City University of New York, New York, NY 10010, USA; youngdeok.hwang@baruch.cuny.edu

**Keywords:** scoliosis, children with neurological disorders, pelvic obliquity, GMFCS

## Abstract

(1) Background: scoliosis is highly prevalent in children with neurological disorders, however, studies predicting the progression and affecting the direction of scoliosis have been insufficient. We investigated the factors associated with the progression and direction of scoliosis in children with neurological disorders. (2) Method: retrospectively, 518 whole spine radiographs from 116 patients were used for analysis. Factors affecting the progression of scoliosis over time were analyzed using linear mixed-effects model. Factors associated with the apex direction of the scoliosis were analyzed. (3) Results: pelvic obliquity (PO) ≥ 2.5°, gross motor function classification system level V, vertebral rotation, and female sex significantly affect the progression of scoliosis (*p* = 0.04, <0.001, <0.001, 0.005, respectively). The higher side of PO and the apex side of scoliosis were interrelated (χ² = 14.58, *p* < 0.001), but the asymmetrical neurological upper extremity involvement was not. (4) Conclusions: severely impaired gross motor function, PO, vertebral rotation, and female sex were significantly related to the progression of scoliosis. The higher side of PO was opposite to the apex side of scoliosis. By identifying the factors that influence the progression of scoliosis, patients at high risk could be more actively intervened to minimize the severe complications.

## 1. Introduction

Scoliosis is defined as a structural alteration of the spine, in which the coronal plane curvature of the spine is greater than 10°. It is known that 25–74% of patients with cerebral palsy (CP) and 48–93% of patients with Duchenne muscular dystrophy have scoliosis [1]. In a previous study [2] of CP patients with an average follow-up period of 16.5 years, the mean age of onset of scoliosis was 6.6 years, and about 50% of patients developed before six years of age. In this study, remeasurement after age 20 showed that 32.5% of patients had a scoliosis progression greater than 10°. This rapid progression of scoliosis has drawn attention to factors influencing the progression of scoliosis in children with neurological disorders, defined as disorders that involve central and peripheral nervous system.

Despite the various problems such as difficulty in maintaining seating and standing postures, pain, skin integrity, and cardiopulmonary dysfunction caused by scoliosis in these patients, the factors that predict scoliosis progression and determine the direction of the apex of scoliosis have been inconclusive. A systematic review in children with severe CP in 2010 found no definite evidence of an association between a few variables such as age, type of CP, location of a scoliosis curve, pelvic obliquity (PO), hip dislocation, and progression of scoliosis [3]. In 2011, Gu et al. analyzed 110 patients with non-ambulatory spastic tetraplegic CP and found that Cobb angle greater than 40° by age 12 was a significant predictor of scoliosis progression [4]. In 2018, Yoshida et al. investigated the natural history of scoliosis in 113 patients with CP and found the risk factors for progression of scoliosis were hip joint displacement, early onset of scoliosis, and Cobb angle of 30° before the age of 10 [2].

Few previous researchers investigated the factors determining the apex side of scoliosis. Hägglund et al. [5] analyzed 79 patients with CP with a PO greater than 5°, suggested associations between the convexity of the scoliotic curve and the higher side of the PO, reduced range of hip abduction, and higher migration percentage of the hip displacement. Furthermore, Porter et al. [6] suggested a significant association between the direction of the apex of scoliosis and the windswept hip deformity. However, to the best of our knowledge, clinical factors such as hand dominance or asymmetric neurological upper extremity involvement have not been investigated as possible associative factors for the direction of the scoliotic curve.

The primary aim of this study is to determine various clinical and radiological factors that affect the progression of scoliosis in children with neurological disorders. The secondary aim is to identify the direction of scoliosis and its relationship to PO, asymmetric upper extremity function, and handedness.

## 2. Materials and Methods

### 2.1. Study Design and Participants

A retrospective observational study was conducted in children or adolescents from the department of rehabilitation medicine at St. Vincent’s hospital between January 2005 and December 2020. A pediatric physiatrist verified the diagnosis of a neurological condition. Among them, patients under 20 years of age with scoliosis were confirmed on the first X-ray, and who had taken anteroposterior (AP) and lateral whole spine radiographs at least 3 times during the follow-up period were recruited. Of the 137 selected subjects, 17 subjects without neurological disorders such as juvenile idiopathic scoliosis were excluded. Overall, 120 subjects and 527 radiographs were taken for measurement. Among the selected X-rays, four images from two subjects were unmeasurable due to pacemaker and brace. Three images from one subject were excluded due to screw fixation of the spine, and two images from one subject were excluded due to poor resolution. Finally, 116 patients were included, and 518 adequate longitudinal scoliosis radiographs were available for analysis. Cobb angle, PO, lumbar lordosis, and vertebral rotation were measured by two physiatrists (J.P. and L.J.). Gross motor function, sex, seizure history, asymmetricity of upper extremities, and handedness were reviewed based on medical charts. Gross motor function was measured with the gross motor function classification system (GMFCS), with levels from I (independent self-ambulation) to V (total dependent mobility, incomplete head control), at the initial evaluation [7]. The asymmetric neurological function of upper extremities was defined as the difference of one or more grades of motor power or spasticity between the right and left sides of upper extremities in terms of manual muscle test or modified Ashworth scale. The handedness was determined to be mainly used side for eating, reaching, grasping, and writing. It was classified as ‘unknown’ when it was difficult to determine the degree of upper extremity function or handedness through chart review.

### 2.2. Measurement Parameters

#### 2.2.1. Scoliosis

**Figure 1:**
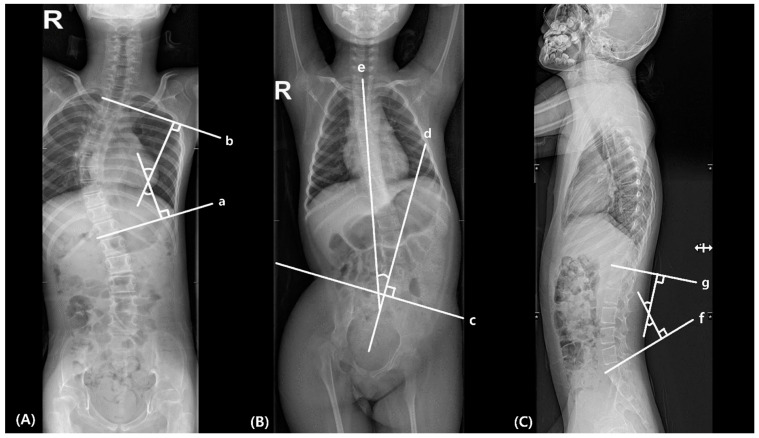
Measurement of (**A**) scoliosis, (**B**) pelvic obliquity and (**C**) lumbar lordosis. (a) The inferior endplate of the lowermost vertebral body in scoliosis curvature; (b) the superior end plate of the uppermost vertebral body in scoliosis curvature; (c) line connecting the bilateral iliac crests; (d) perpendicular line to the line connecting the bilateral iliac crests; (e) line between the center of T1 to S1 vertebral bodies; (f) S1 superior endplate line; (g) T12 inferior endplate line.

Scoliosis was measured by Cobb’s method, the angle between the superior end plate of the uppermost vertebral body and the inferior end plate of the lowermost vertebral body in scoliosis curvature in the AP radiographs (Figure 1A) [8]. The coronal direction of scoliosis was determined by the apex, defined as the farthest vertebrae from the central sacral vertical line, a vertical line perpendicular to the line across the top of the iliac crests [9]. Thus, the direction of scoliosis curve was determined by the direction of the apex.

#### 2.2.2. Pelvic Obliquity (PO)

PO was measured with the Maloney method, defined as the angle between the line perpendicular to the line connecting the bilateral iliac crests and the line between the center of T1 to S1 vertebral bodies in the AP radiographs (Figure 1B) [10]. The coronal direction of the PO was determined by the relationship between the two lines above. PO was also measured related to the presence of scoliosis.

#### 2.2.3. Lumbar Lordosis

Lumbar lordosis was measured in the lateral radiographs by Cobb’s method, angle between S1 superior end plate and T12 inferior end plate (Figure 1C) [11].

#### 2.2.4. Vertebral Rotation

All AP radiographs were assessed vertebral rotational deformity by Nash–Moe method, defined as the percentage displacement of the convex pedicle in relation to the vertebral body width [12].

### 2.3. Statistical Methods

Descriptive analysis was used for all variables, including median and interquartile range for continuous data and percentages for categorical data. Inter-rater reliability of radiographic indices was assessed by interclass correlation coefficients (ICCs) estimates, and their 95% confident intervals (CI) based on a mean-rating (k = 2), absolute-agreement, 2-way mixed effects model [13]. A Chi-squared test was performed to evaluate the association between the apex side of scoliosis and handedness, upper limb function asymmetry, and PO. Statistical tests for ICCs, patient’s demographics, clinical characteristics, and factors affecting the apex side of scoliosis were performed using SPSS (ver. 21.0; SPSS, Chicago, IL, USA). To assess the progression of scoliosis as measured by Cobb angle with age, we used a linear mixed-effects model (LMM) with a restricted maximum likelihood method for parameter estimation, with random intercept and slope with the possible correlation between them. Each factor affecting the progression of scoliosis (PO, GMFCS level, lumbar lordosis, vertebral rotation, sex, and history of epilepsy) was considered a fixed effect, and each patient was considered a random effect. Statistical tests for factors affecting the progression of scoliosis were performed using MATLAB Release 2021a and Statistic and Machine Learning Toolbox (MathWorks, Natick, MA, USA). *p* values less than 0.05 were considered statistically significant.

## 3. Results

The patient’s demographics and clinical characteristics are shown in Table 1. Fifty-five patients (47.4%) were female. The patient’s median (interquartile range) age at the initial evaluation was 3.1 (1.7–5.9) years, and at the last assessment was 18.05 (11.34–21.91) years. The median (interquartile range) duration of the interval between each radiograph was 1.11 (0.75–1.91) years, and the period between initial and last radiographs was 5.0 (2.6–8.4) years. The number (percentage) of patients over 18 years of age at the initial evaluation was zero (0%) and at the last assessment was five (4.31%). Cobb angle greater than 10° was observed in 45 patients (38.79%) at the initial evaluation and 68 patients (58.62%) at the last evaluation. Most of the patients had cerebral palsy (107, 92.2%), and others had brain neoplasm (2), Fukuyama muscular dystrophy (1), spinal muscular atrophy (1), mitochondrial diseases (1), Rett syndrome (1), Lesch Nyhan syndrome (1), Dandy–Walker syndrome (1), and Noonan syndrome (1). During the follow-up period, two patients applied thoraco-lumbo-sacral braces, thirty-seven non-ambulatory patients used seating system, and seven patients underwent hip joint surgery due to hip dislocation (five bilaterally, two left side).

Inter-rater reliability of the measurements is described in Table 2, with good reliability for PO and vertebral rotation and excellent reliability for scoliosis Cobb angle and lumbar lordosis angle [13]. The difference in mean angle of scoliosis, PO, and lumbar lordosis between the two inspectors were 3.88, 2.42, and 7.78 degrees, respectively.

### 3.1. Factors Affecting the Progression of Scoliosis

**Figure 2:**
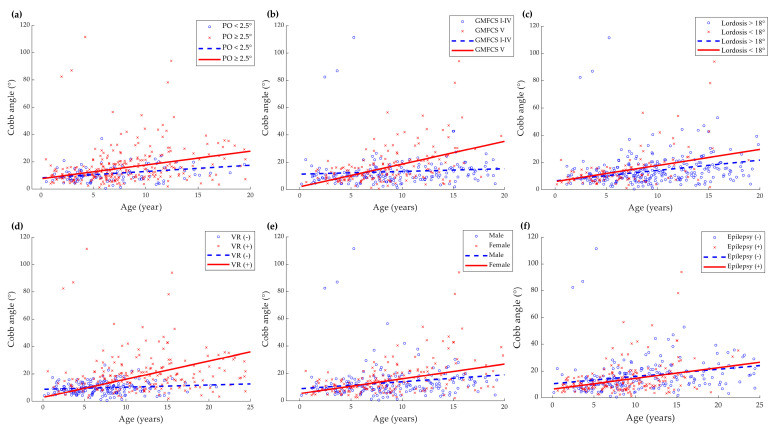
Subject-specific (scatterplot) and predicted progression line of Cobb angle (°) versus age (years) according to clinical parameters: (**a**) pelvic obliquity (PO); (**b**) gross motor function classification system (GMFCS); (**c**) lumbar lordosis; (**d**) vertebral rotation (VR); (**e**) sex; (**f**) epilepsy. There was a significant increase in the progression rate of scoliosis in patients with PO ≥ 2.5°, GMFCS level V, vertebral rotation (Nash–Moe grade ≥ 1), and in female patients. Reduced lumbar lordosis (<18°) in ≥2 years of age and the presence of epilepsy did not significantly affect scoliosis progression.

Figure 2 shows the subject-specific profiles and estimated annual change of Cobb angle according to each factor (PO, gross motor function, lumbar lordosis, vertebral rotation, sex, and epilepsy). PO greater than 2.5°, GMFCS level V, the presence of vertebral rotation (Nash–Moe grade ≥ 1), female sex were significant predictors of the progression of the scoliosis curve (*p* = 0.04, <0.001, <0.001, 0.005, respectively). Reduced lumbar lordosis (less than 18°) at or more than two years of age and the presence of epilepsy did not significantly contribute to the progression of scoliosis (*p* > 0.05 in both). A value that is two standard deviations lower than the average value of typically developing children aged 2–4 years reported in the previous study was used as the reference for reduced lumbar lordosis [14].

### 3.2. Factors Affecting the Apex Side of Scoliosis

The effects of handedness and asymmetric upper limb function were analyzed in 82 patients with a Cobb angle of 10 degrees or more based on the final radiograph of each patient. Among them, the effect of PO was analyzed in 58 patients with 2.5 or more degrees of PO. The Chi-square test (Table 3) revealed that functional asymmetricity of upper limbs and handedness was not significantly associated with the apex side of scoliosis (χ² = 1.87 and 4.21, respectively, *p* > 0.05). However, in patients with PO ≥ 2.5°, there was a significant correlation between the higher side of the PO and the apex side of the scoliosis (χ² = 12.91, *p* < 0.001). The proportion of patients with apex side of scoliosis opposite to the high side of PO was 75.0% (43/58). Subgroup analysis was performed to investigate the correlation between the secondary curve apex and the higher side of PO. The secondary curve was at the lumbar level in six out of fifty-eight patients and at the thoracic level in ten patients. In the group with the secondary curve at the thoracic level, the higher side of PO was located on the opposite side of the major curve apex in eight out of ten patients (80.0%), but in the group with the secondary curve at the lumbar level, one out of six patients (16.7%), the higher side of PO was located on the opposite side of the major curve apex. Though it was difficult to confirm the statistical significance due to the small number of patients, the presence of secondary curve at the lumbar level tended to show additional associations with the higher side of PO.

## 4. Discussion

Among spinal deformity, adolescent idiopathic scoliosis is the most common deformity, occurring in healthy children around puberty and progressing with skeletal growth. In patients with a curve of less than 30° after bone maturation, scoliosis rarely progresses, and in the case of 30° to 50°, an average of 10° to 15° progresses throughout lifetime [15]. On the other hand, neuromuscular scoliosis can occur even before puberty, and in previous study, there was no significant decrease in the progression of scoliosis after growth maturation, and the Cobb angle could continuously increase [2]. Saito et al. [16] observed that 11 out of 13 (85%) had a spinal curve of 40° or more at the age of 15, and as a result, the curvature progressed to more than 60° in patients with cerebral palsy. According to the aggressive nature of the progression, neuromuscular scoliosis causes more physiologic impairment affecting gait, sitting posture, pain, and decreased cardiopulmonary function than adolescent idiopathic scoliosis.

In this study, we found that increased PO, severe motor dysfunction (GMFCS level V), vertebral rotation, and female sex were contributing to the progression of scoliosis with age. In general, an ambulatory function is known as a critical factor for scoliosis [17]. Several investigators have identified GMFCS as a decisive factor influencing scoliosis progression, which supports our results. Lee et al. [18] estimated the annual changes in radiographic indices of the spine in 184 patients with CP and found that the annual increase in scoliosis Cobb angle was higher in the GMFCS Level IV–V group than in the GMFCS Level I–II and III groups. Garg et al. [19] analyzed the difference in scoliosis progression according to GMFCS level in 115 CP patients who underwent varus derotational osteotomy surgery. The authors reported that the annual progression in the GMFCS I–III and IV groups was 1°–2° degrees, but in the GMFCS V group, the annual progression was 3.5°, showing more rapid progression. Similar results were obtained in this study as well. With the analysis by GMFCS level, there was no significant difference in GMFCS Level I–IV. However, the slope in Cobb angle in GMFCS Level V was significantly different from other levels.

Previous researchers have investigated the association between coronal spine deformities and PO in scoliosis [5,19]. Biomechanically, the pelvis serves as an important pivot between the trunk and lower extremities, playing a fundamental role in maintaining an unsupported sitting posture and ambulation [20]. Therefore, the progression of scoliosis with PO and the loss of the sitting balance are common features in severely affected patients with neurological disorders. A recent study reported that the progression of scoliosis, PO, and hip subluxation were related to each other in non-ambulatory patients with neuromuscular disorders, however, the sequence of development was difficult to determine [21]. Generally, PO more than 5 degrees is regarded as significant; however, even 2.5 degrees of PO was revealed to be significantly related with the progression of Cobb angle in this study. Further studies are needed on the biomechanical role of PO and the sequence of structural alterations in the hip, pelvis, and spine.

Vertebral rotation is an essential component of scoliosis from a three-dimensional perspective. Mohanty et al. [22] analyzed 158 patients with adolescent idiopathic scoliosis and revealed a significant association between Cobb angle and Nash–Moe grade. Evidence for the role of vertebral rotation in scoliosis is limited. Although the presence of vertebral rotation had a significant effect on the progression of scoliosis in our results, the effect of the degree of rotation could not be confirmed as the number of patients with Grade 2 or higher vertebral rotation in the Nash–Moe grade was small.

Female sex is known to be a factor influencing the progression of the curve in adolescent idiopathic scoliosis [15]. In the result of our study, the slope of the trend line was significantly larger in girls, showing a similar trend as in idiopathic scoliosis. In patients with neurological disorders, the female sex has been suggested to increase the risk of developing scoliosis, and the odds ratio for females was 1.9 compared to males [23,24]. However, for the progression of scoliosis, sex did not have a significant effect in the previous study [18], further research involving a large sample size is required.

Previous studies have also suggested the relationship between the development of scoliosis and epilepsy [23,24]; however, no significant effect on progression was identified in our result. Whether epilepsy itself is a risk factor for scoliosis or whether children with epilepsy tend to have more severe functional disabilities is unclear. Moreover, the effect of epilepsy on the progression of scoliosis is inconclusive.

A secondary analysis of the factors determining the apex side of scoliosis demonstrated that PO was a significant factor; however, handedness or asymmetric upper limb function had no significant effect on the direction of scoliosis. The current results were consistent with the previous reports that 89% of the patients with CP had the convexity of scoliosis on the opposite side of the high side of the PO [5]. We additionally found that this tendency was offset in the case of patient whose secondary curve was at the lumbar level. In conclusion, pelvis is thought to serve as a critical bone that determines the direction and influence the progression of the scoliotic curve.

A limitation of our study is that since all data were collected retrospectively, an unequal number of radiographs were collected for each subject at non-fixed time points. However, to compensate for this problem, we used the LMM to determine whether each variable significantly influenced the trend of changes in Cobb angle with time [25]. Second, we did not measure the location or type of scoliotic curvature, the severity of hip subluxation or hip dislocation, history of hip surgery, or decreased range of hip and knee joints motion. These may be another possible factor influencing scoliosis progression, and further studies including these factors are needed. Third, since the data were collected in rehabilitation clinic, the severity of scoliosis tends to be milder than that of other studies, even with severe gross motor function. Children with more severe scoliosis were referred to an orthopedic surgeon and thereafter were not included in this data. In addition, many of them are receiving rehabilitation therapy and may be a group with a high level of interest from the caregiver. Forth, the median duration of follow-up was 5 years, which may miss the progressive stage of scoliosis in some patients. Fifth, Rett syndrome and Lesch Nyhan syndrome are gender-specific syndromes involving female and male, respectively. However, only one patient for each syndrome was included in this study, which may have negligible impact on the analysis of gender effects on scoliosis progression. Lastly, GMFCS was applied to measure the gross motor function of all children including patients other than CP. Although GMFCS has been validated for children with CP and Down syndrome, it has been widely used to measure gross motor function in children with motor difficulties [26,27].

Various conservative treatments have been tried to prevent the progression of scoliosis in patients with neurological disorders. Physiotherapy and spinal orthoses are helpful for muscle balance, postural support, and prevention of contractures but are known to have a limited role in decreasing curve progression [28,29]. An intensive pharmacological method such as an intrathecal baclofen pump was revealed not to change the progression of scoliosis with CP [30]. Although observation and non-surgical treatment are recommended when the curve is less than 40 degrees, most scoliosis results in spinal instrumentation and fusion due to significant curve progression, loss of sitting balance, and function [31]. By identifying the factors that influence the progression and apex direction of scoliosis, patients at high risk could be more actively intervened, such as shortening the follow-up period or preemptively referring to a surgeon. Future research that proposes predictive algorithms for scoliosis progression and provides criteria for distinguishing groups that require active intervention is needed to use medical resources more efficiently while benefiting patients.

## 5. Conclusions

Our results indicated that PO, GMFCS Level V, vertebral rotation, and female sex were important factors affecting the progression of scoliosis. Underdevelopment of lumbar lordosis and epilepsy had no significant effect on the progression of scoliosis. In addition, there was a significant association with PO and scoliosis curve direction, with the higher side of PO was opposite to the apex side of scoliosis. However, asymmetric upper limb function or handedness did not affect the direction of scoliosis. The factors identified in our study can be used for further studies on the predictive model of scoliosis progression in patients with neurological disorders.

## Figures and Tables

**Table 1:** Participants’ demographic data.

Parameter	Value, N (Range or %)
Subjects	116
Female	55 (47.4)
Age at the first radiograph (years) ^1^	3.1 (1.7–5.9)
Age at the last radiograph (years) ^1^	18.05 (11.34–21.91)
Interval between each radiograph (years) ^1^	1.11 (0.75–1.91)
Period between initial and last radiographs (years) ^1^	5.0 (2.6–8.4)
Handedness, right/left/unknown	38/34/44 (32.8/29.3/38.0)
Functional asymmetry of upper limbs, yes/no/unknown	56/47/13 (48.3/40.5/11.2)
Presence of epilepsy	51 (44.0)
GMFCS ^2^ level	
I	7 (6.4)
II	14 (12.7)
III	6 (5.5)
IV	37 (33.6)
V	46 (41.8)

^1^ Median (interquartile range), ^2^ GMFCS; gross motor function classification system.

**Table 2:** Inter-rater reliability of radiographic measurements.

Measurements	ICC ^1^	95% CI ^2^
Scoliosis Cobb angle (°)	0.955	0.945–0.964
Pelvic obliquity (°)	0.840	0.804–0.870
Lumbar lordosis angle (°)	0.929	0.906–0.945
Vertebral rotation (Nash-Moe grade)	0.807	0.755–0.847

^1^ ICC; interclass correlation coefficient, ^2^ CI = confidence interval.

**Table 3:** The direction of apex of scoliosis and clinical parameters (functional asymmetry of upper limbs, handedness, and higher side of pelvic obliquity).

		Apex of Scoliosis, N (%)			*p* Value
		Right	Left	Total	
Functional asymmetry of upper limbs	Yes	13 (33.3)	26 (66.7)	39 (100.0)	0.39
	No	14 (43.8)	18 (56.3)	32 (100.0)	
	Unknown	6 (54.5)	5 (45.5)	11 (100.0)	
Handedness	Right	6 (54.5)	5 (45.5)	11 (100.0)	0.12
	Left	13 (41.9)	18 (58.1)	31 (100.0)	
	Unknown	4 (21.1)	15 (78.9)	19 (100.0)	
Higher side of pelvic obliquity	Right	13 (28.9)	32 (71.1)	45 (100.0)	<0.001 *
	Left	11 (84.6)	2 (15.4)	13 (100.0)	

* Significant correlation (*p* < 0.05).

## Data Availability

The data presented in this study are available on request from the corresponding author.
